# Pyocystis Causing Abdominal Wall Abscess and Necrotising Fasciitis

**DOI:** 10.7759/cureus.79938

**Published:** 2025-03-02

**Authors:** Simone Sim, Theodora Stasinou

**Affiliations:** 1 Department of Urology, Manchester Royal Infirmary, Manchester, GBR

**Keywords:** abdominal wall abscess, benign urology, case report, defunctioned bladder, necrotising fasciitis (nf), pyocystis complication, urinary tract infection

## Abstract

Pyocystis is a rare but known complication of the defunctioned bladder. We report a case of a female patient with long-term bilateral nephrostomies who was initially thought to have cystitis but later found to have pyocystis causing a vesicocutaneous fistula and an anterior abdominal wall abscess. It progressed to necrotising fasciitis, necessitating emergency drainage and debridement. This yielded 400 ml of pus, and she was found to have a defect in the anterior rectus sheath leading straight to the bladder. This report highlights the importance of maintaining a high index of suspicion for pyocystis in patients with a defunctioned bladder, and how delays in diagnosis and treatment can cause significant morbidity.

## Introduction

Pyocystis, also known as empyema cystitis or vesical empyema, is a rare but severe lower urinary tract infection seen in patients with a defunctioned bladder. It is caused by an accumulation of mucosal debris in the bladder with a secondary infection. Patients with defunctioned bladders include those with suprapubic urinary diversion with remnant bladder, bilateral nephrostomies, or anuric patients with end-stage renal disease [1]. The reported incidence of pyocystis in the adult population with a defunctioned bladder from suprapubic urinary diversion ranges from as low as 3.3% [2], to as high as 67% in those with previously irradiated bladders [3].

The presenting symptoms for pyocystis are similar to those in lower urinary tract infections - fever, suprapubic pain, purulent urethral discharge, and urosepsis in severe cases. It is an often-forgotten source of infection in those at-risk groups mentioned above as it could be mistaken for simple cystitis [4]. It is also not uncommon for female patients with defunctioned bladders to be misdiagnosed with vaginal discharge instead [5]. As such, pyocystis can be difficult to detect, leading to delays in appropriate treatment and, consequently, increased morbidity.

Given the rarity of the disease, there are no formal guidelines for the treatment of pyocystis. However, the most common first-line approaches from reported cases include catheterisation, intravesical irrigation, and administration of antibiotics [1]. This report discusses a rare case of pyocystis that had progressed to form an anterior abdominal wall abscess and subsequently necrotising fasciitis.

## Case presentation

A female in her 60s with a background of permanent bilateral nephrostomies in situ for bilateral ureteric strictures secondary to urolithiasis presented to the emergency department with a two-week history of lower abdominal pain, pyrexia and cloudy urine output from her right nephrostomy. Her family doctor had initially treated her for suspected cystitis with a seven-day course of oral pivmecillinam, but her symptoms had worsened. Due to the nephrostomies, all urine was completely diverted from the bladder and she did not pass any urine per urethra. She had ongoing discharge that had been unchanged for weeks, but unsure if per urethra or vagina. Her medical history included stage four chronic kidney disease with a left arm arteriovenous fistula (but she had yet to start dialysis); emphysema; ischaemic heart disease; and insulin-dependent type two diabetes mellitus. On examination, her abdomen was slightly distended and tender in the suprapubic region.

Investigations

**Figure 1:**
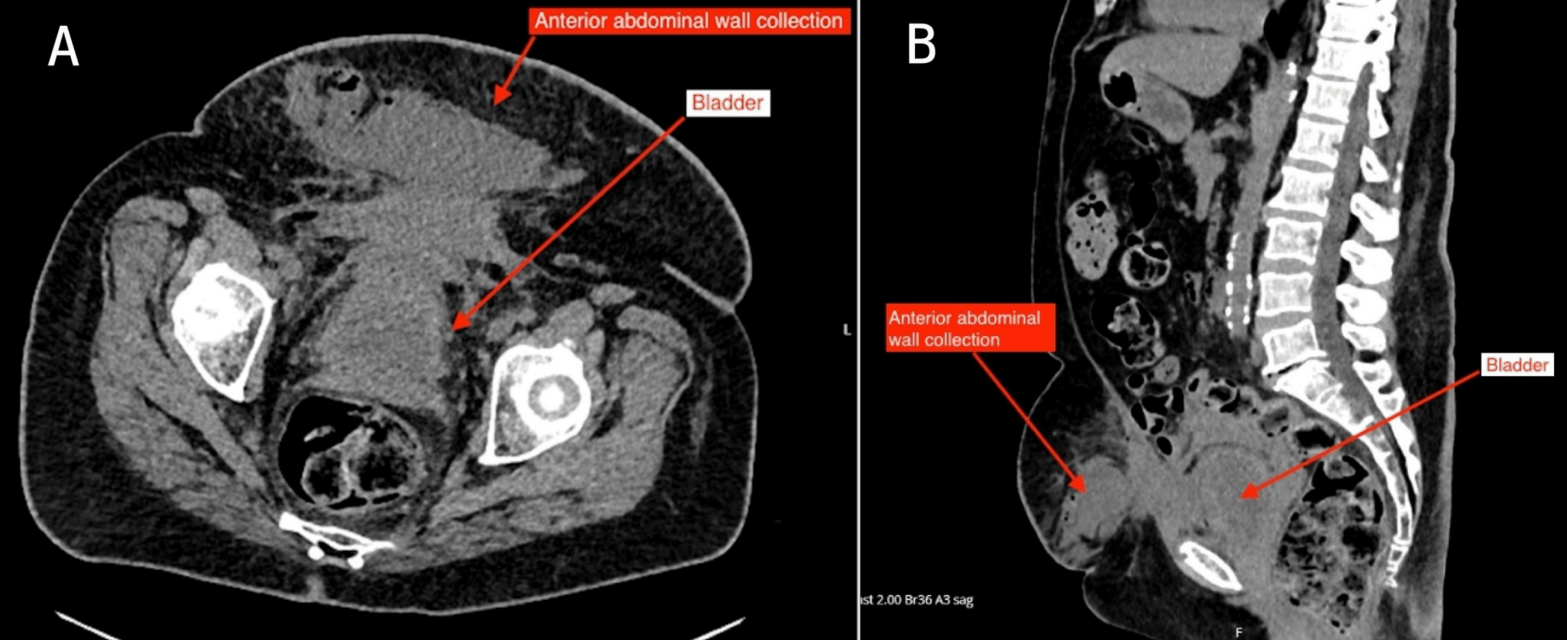
Axial (A) and sagittal (B) views of non-contrast CT abdomen and pelvis A large collection within the subcutaneous fat of the lower abdomen measuring 18 x 4.3 x 6 cm was noted. This was in continuity with the anterior abdominal wall muscles with inflammatory stranding of the intra-abdominal fat extending onto the bladder. The lack of intravenous contrast made it difficult to confirm the involvement of the anterior bladder wall CT: computed tomography

She was admitted under the medical team for intravenous (IV) antibiotics for the treatment of cystitis. After five days of IV temocillin, she remained apyrexial, haemodynamically stable, and her inflammatory markers showed marginal improvement (Table 1). She was also draining clear urine from both nephrostomies. Hence, she was switched to oral co-amoxiclav. However, her abdominal pain persisted. The next day, she started to spike temperatures of 38.3 °C; she was tachycardic with a heart rate of 110 bpm, and her inflammatory markers began to rise again (Table 1). Clinically, she was tender in her lower abdomen, and a palpable fullness was noted. This prompted an urgent CT scan of her abdomen and pelvis. Given the state of her renal function, a non-contrast scan was done (Figures 1A, 1B).

**Table 1:** Patient's laboratory result trends Marginal improvement of inflammatory markers (WCC and CRP) was observed from Day 0 to 5 with IV temocillin. There was a marked rise in CRP and WCC on Day 6 in keeping with her clinical deterioration. Persistently low renal function (eGFR) in keeping with her known chronic kidney disease was also noted

Blood test	Reference range	Day 0	Day 1	Day 4	Day 5	Day 6
White blood cell count (WCC)	4.0–11.0 x10^9/L	23	18	13	13	20
C-reactive protein (CRP)	0-5 mg/L	346	273	293	290	362
Estimated glomerular filtration rate (eGFR)	>90 mL/min/1.73m^2^	14	13	14	15	15

Treatment and outcome

The patient was reviewed by the local General Surgery team in light of the CT findings. On examination, her lower abdomen was mildly distended and tender with a palpable swelling, but no crepitus or skin changes. Given the proximity of the collection and degree of fat stranding extending to the bladder, there were concerns that the bladder was communicating with the abscess. However, due to the CT being a non-contrast scan, it was difficult to confirm this. While a urethral catheterisation that would help determine the nature of and drain the contents in the defunctioned bladder was deemed necessary, the patient had refused this in the past due to pain and intolerance to the procedure. Additionally, this prevented a CT cystogram from being done, which could have helped to delineate any bladder defects. As there was no weekend Interventional Radiology (IR) service in the local hospital, the case was discussed with the staff at the tertiary hospital where Urology and IR were on-site. She was accepted for urgent transfer to the tertiary hospital for ultrasound-guided drainage of the abscess.

**Figure 2:**
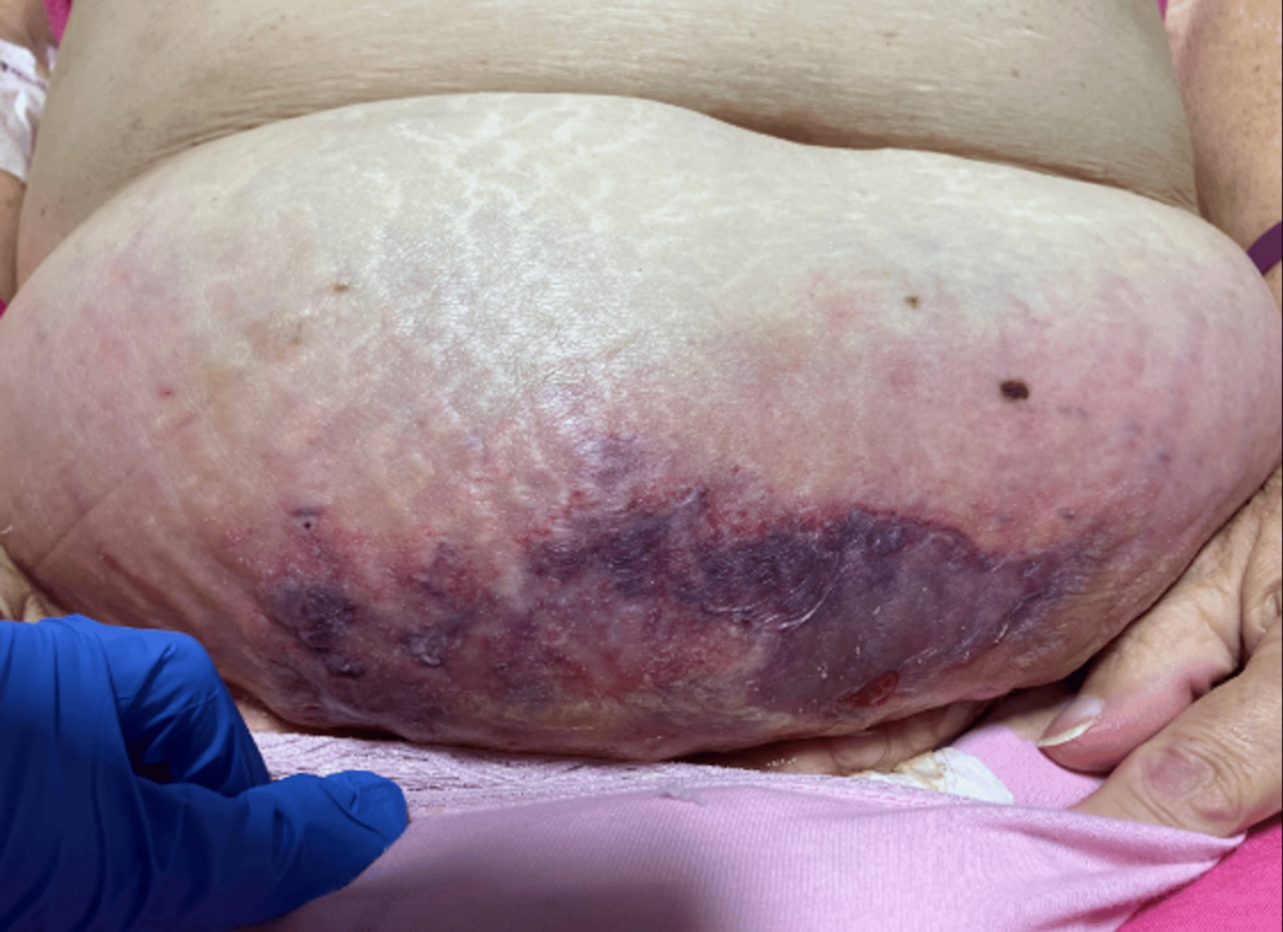
Photo of patient’s abdomen On arrival to the tertiary hospital, a large area of ecchymosis was noted

On arrival at the tertiary centre, it was noted that an area of ecchymosis had developed and progressed rapidly within a few hours (Figure 2). The General Surgery team was alerted, and they agreed that the appearances were in keeping with necrotising fasciitis. She was taken to the theatre for a debridement of the collection; 400 ml of pus was drained from the anterior abdominal wall, and a 1 cm hole in the anterior sheath was found, leading directly into the bladder. The Urology consultant on call was called to the theatre, who placed a 12 Fr catheter via this defect, and a further 14 Fr urethral catheter. The necrotic tissue was debrided back to healthy bleeding tissue. The cavity was left open, packed with betadine-soaked gauze, and secured with Mefix dressing.

After the procedure, the patient was admitted to the ICU. Three days after the operation, a joint decision was made between the Urology, General Surgery, and tissue viability team to re-explore the wound and attempt a primary closure to aid wound healing. The size and location of the wound would make healing from secondary intention difficult. For her re-look operation, further dead tissue was debrided. The abdominal catheter was removed, and the bladder defect was suture-repaired in two layers. A cystoscopy was also conducted which showed a slough in the posterior bladder wall. This was biopsied and sent for histological analysis. The abdominal wound was approximated and repaired in layers, and the wound was closed.

The pus sample taken on the initial drainage grew *Enterococcus spp.*, which was resistant to amoxicillin but sensitive to vancomycin. This positive culture was also present on the initial nephrostomy urine culture taken on admission. The bladder biopsy showed dense neutrophilic infiltrate and features in keeping with severe acute inflammation or ulcer slough.

The patient continued to improve in the ward, with regular reviews from the wider multi-disciplinary team including tissue viability nurses, dieticians, and physiotherapists. She was eventually discharged, and to date, she is still having regular district nurse reviews in the community for wound management. Since this encounter, she has been placed on a long-term catheter with regular flushes to prevent the re-accumulation of pus.

## Discussion

Pyocystis is the most common complication arising from the defunctioned bladder [3]. It is a clinical entity that gained traction in the 1960s after suprapubic urinary diversion (ileal conduit formation) was also used to treat benign pathologies, as concomitant cystectomy was not routinely performed [6]. Most retrospective analyses focus on patients who had suprapubic urinary diversion with a remnant bladder. However, it is important to keep in mind that the cohort of patients with a defunctioned bladder also includes those who have the diversion of urine via bilateral nephrostomies, and physiologically defunctioned bladder secondary to anuria in end-stage kidney disease [7,8].

The pathogenesis of pyocystis involves the accumulation of exfoliated urinary bladder epithelium that liquefies and becomes infected when the bladder is not evacuated. In urinary diversion for benign pathologies, the bladder is disconnected from the urinary system but still has an intact blood and nerve supply which produces mucosal debris. In patients with end-stage renal failure, oliguria and anuria create inadequate washout of the urinary bladder due to low flow in the urinary system [8].

Patients with a defunctioned bladder are at an inherent risk of pyocystis due to the pathophysiological changes to the bladder when it becomes defunctioned. Although there is limited data, it is recognised that the defunctioned bladder atrophies, and its capacity and compliance decrease significantly [9]. Functional and pharmacological studies on the canine bladder with urinary diversion have shown reduced intravesical capacity in 47% of controls at six months, reduced compliance, reduced contractility to muscarinic stimulation, and a decrease in muscarinic receptor density [10]. Fortunately, evidence from human studies shows that these changes can be reversed by undiversion [9]. This explains the inability to expel mucosal debris in defunctioned bladders even when there is no concurrent bladder outlet obstruction. There are additional risk factors for pyocystis, such as previous pelvic radiation, concurrent bladder outlet obstruction (i.e., stricture), chronic infection, and history of *Proteus* urinary tract infection [2]. In our patient, there is a possibility of an additional neurogenic component to her poor bladder-emptying given her history of diabetes mellitus, though it has not been formally investigated.

A diagnosis of pyocystis can be challenging in patients who do not pass urine per urethra. Our patient was initially diagnosed as a case of simple cystitis. The fact that she was producing good amounts of clear urine from the bilateral nephrostomies could have been falsely reassuring, and urethral catheterisation was not suggested early in her clinical course. It was only when the patient failed to show improvement on IV antibiotics five days later that further investigations in the form of a non-contrast CT scan were done, which revealed the abscess. This anterior abdominal wall abscess was also missed clinically due to the lack of skin changes and the large body habitus of the patient.

The CT scan showing the proximity of the abscess to the bladder showed the need for urethral catheterisation, which the patient refused due to pain and intolerance to the procedure in the past. A CT cystogram would have helped delineate any defects of the bladder wall, but it could not be done without a catheter. The value of a CT urogram was questionable, and relatively contraindicated due to the poor excretory function of her kidneys. Overall, the patient was initially misdiagnosed as a case of simple cystitis, which caused delays in recognising the need for urgent drainage of the bladder. Furthermore, once the anterior abdominal wall abscess was noted, it was difficult to confirm that the source of the abscess was the bladder.

There are no formal guidelines for the treatment of pyocystis due to the rarity of the disease. However, the most common approaches from reported cases include catheterisation, intravesical irrigation, and administration of antibiotics [1,4,9]. Once pyocystis is confirmed, blood and urine cultures should be collected. Resuscitation and sepsis protocols should be followed if indicated. Antibiotics with gram-negative bacteria cover should be started according to local antimicrobial guidelines, taking into account the patient’s historical urine culture and sensitivities. Urethral catherisation is important to drain the intravesical pus, and bladder irrigation or manual washouts with either normal saline or bactericidal solutions are recommended [1]. Surgery in the form of cystectomy or a Spence-Allen procedure (creation of a fistula between a defunctioned bladder and the anterior vaginal wall to allow permanent bladder drainage [9]) should be considered in patients with minimal response to conservative management or those with recurrent moderate to severe pyocystis [1]. In this case, due to the extension of the abscess into the anterior abdominal wall, emergent intervention in the form of abdominal debridement was required.

## Conclusions

Physicians should maintain a high index of suspicion for pyocystis in patients with anatomical or physiological defunctioned bladder, who have lower urinary tract infections. Urethral catheterisation is vital for the diagnosis and management of pyocystis to allow for the timely drainage of the intravesical abscess, and delays in treatment can cause significant disease progression and morbidity.
